# 9 Challenges Related to BICU Nurses: The Burden of the Burn Unit

**DOI:** 10.1093/jbcr/iraf019.009

**Published:** 2025-04-01

**Authors:** Landon Oquinn, Anastasiya Ivanko, Shana Lennard, Jonathan Schoen, Jeffrey Carter

**Affiliations:** Burn Center at University Medical Center; Burn Center at University Medical Center; Louisiana State University Burn Center; Louisiana State University Health Sciences Center - New Orleans; Louisiana State University Burn Center

## Abstract

**Introduction:**

The importance of a multidisciplinary approach in the treatment of a burn patient cannot be overstated. The demands of these critically ill patients are high, thus aggravating the physical and emotional exhaustion of staff registered nurses (RNs). Burns is a highly intensive field, and nurses form the largest subgroup of the burn care team. Efforts into understanding the wellness of burn nurses must be taken to preserve the specialized care team of the burn population.

**Methods:**

After obtaining approval from the Institutional Review Board (IRB), we conducted a de-identified survey regarding burn nurse wellness at our American Burn Association Verified Burn Center. Solicitation to participate and data acquisition was done via email and Survey Monkey. Continuous and dichotomous variables were analyzed using Microsoft Excel.

**Results:**

Fifteen respondents, predominantly female (73.3%) and aged 30-39, participated. Educational backgrounds varied, with 66.6% holding BSN degrees and 46% CBRN certification. Experience ranged from 0 to 20 years, with 53.3% having 5-10 years in burn care. Majority were not part of a nurse union, and duties included wound care (87.7%), ICU and step-down patient care (73.3%), burn consults (46.7%), clinic (20%), etc. Respondents worked 38.1 hours weekly (±8.47), primarily on day shifts (80%). Burnout symptoms were reported by 46.7%, with 26.7% considering leaving burn care within 2 years and 33.3% in the next 5 years. Satisfaction stemmed from patient recovery and teamwork. Career satisfaction varied, with 60% satisfied yet 79.9% dissatisfied with compensation. Despite feeling respected (80%), balancing personal life was challenging, especially for night-shift RNs. Among parents (33% of respondents), 60% felt they lacked sufficient time off for childbirth and were dissatisfied with parenting responsibilities. Physiological strain, including dehydration (80%), muscular-skeletal injuries (53.3%), and sleep deprivation (86.7%) were commonly reported. Additionally, 46.7% of respondents reported emotional strain from caring for burn patients, indicating deleterious effects on their mental health.

**Conclusions:**

Nursing shortages are a serious global issue, impacting both patient care quality and hospital finances. Our study highlights RN concerns, including burnout symptoms, intentions to leave burn care, satisfaction levels with various career aspects, and the detrimental effects of work-related strain on their physical and mental well-being. Failing to address these challenges may exacerbate the gap between the supply and demand for nursing professionals, risking compromised patient care quality and the long-term sustainability of healthcare systems.

**Applicability of Research to Practice:**

Foster a positive work culture by addressing nurse wellness, which can enhance job satisfaction and ultimately lead to better patient outcomes in burn care.

**Funding for the Study:**

N/A

